# Effect of Sleep Disturbance Symptoms on Treatment Outcome in Blended Cognitive Behavioral Therapy for Depression (E-COMPARED Study): Secondary Analysis

**DOI:** 10.2196/30231

**Published:** 2022-03-21

**Authors:** Esben Skov Jensen, Nicolai Ladegaard, Angelina Isabella Mellentin, David Daniel Ebert, Ingrid Titzler, Ricardo Araya, Arlinda Cerga Pashoja, Jean-Baptiste Hazo, Jérôme Holtzmann, Roman Cieslak, Ewelina Smoktunowicz, Rosa Baños, Rocio Herrero, Azucena García-Palacios, Cristina Botella, Thomas Berger, Tobias Krieger, Trine Theresa Holmberg, Naira Topooco, Gerhard Andersson, Annemieke van Straten, Lise Kemmeren, Annet Kleiboer, Heleen Riper, Kim Mathiasen

**Affiliations:** 1 Centre for Telepsychiatry Mental Health Services of Southern Denmark Odense Denmark; 2 Department of Clinical Medicine Faculty of Health Sciences University of Southern Denmark Odense Denmark; 3 Department of Affective Disorders Aarhus University Hospital Aarhus Denmark; 4 Unit for Psychiatric Research Department of Clinical Research University of Southern Denmark Odense Denmark; 5 Brain Research-Inter-Disciplinary Guided Excellence (BRIDGE) Department of Clinical Research University of Southern Denmark Odense Denmark; 6 Department for Sport and Health Sciences, Chair for Psychology & Digital Mental Health Care Technical University Munich Munich Germany; 7 Department of Clinical Psychology and Psychotherapy Friedrich-Alexander University of Erlangen-Nürnberg Erlangen Germany; 8 Centre for Global Mental Health King's College London London United Kingdom; 9 Department of Population Health London School of Hygiene and Tropical Medicine London United Kingdom; 10 Global Public Health Public Health England London United Kingdom; 11 URC Eco Ile-de-France (AP-HP) Hotel Dieu Paris France; 12 Assistance Publique-Hôpitaux de Paris Health Economics Research Unit University of Paris Paris France; 13 Fondation FondaMental Créteil France; 14 Service de Psychiatrie de l’adulte Centre Expert Dépression Résistante FondaMental Grenoble University Hospital Grenoble France; 15 Faculty of Psychology SWPS University of Social Sciences and Humanities Warsaw Poland; 16 Lyda Hill Institute for Human Resilience University of Colorado Colorado Springs, CO United States; 17 Instituto Polibienestar University of Valencia Valencia Spain; 18 CIBER Fisiopatología Obesidad y Nutrición (CIBEROBN) Madrid Spain; 19 Department of Basic Psychology Clinic and Psychobiology University Jaume I Castellón Spain; 20 Department of Clinical Psychology and Psychotherapy University of Bern Bern Switzerland; 21 Department of Behavioural Sciences and Learning Linköping University Linköping Sweden; 22 Center for m2Health Palo Alto University Palo Alto, CA United States; 23 Department of Biomedical and Clinical Sciences Linköping University Linköping Sweden; 24 Department of Clinical Neuroscience Karolinska Institute Stockholm Sweden; 25 Department of Clinical, Neuro, and Developmental Psychology Vrije Universiteit Amsterdam Netherlands; 26 Amsterdam Public Health Research Institute Amsterdam Netherlands

**Keywords:** blended care, bCBT, cognitive behavioral therapy, digital intervention, major depressive disorder, sleep disturbance, sleep disorder, mental health, digital health, mobile phone

## Abstract

**Background:**

Sleep disturbance symptoms are common in major depressive disorder (MDD) and have been found to hamper the treatment effect of conventional face-to-face psychological treatments such as cognitive behavioral therapy. To increase the dissemination of evidence-based treatment, blended cognitive behavioral therapy (bCBT) consisting of web-based and face-to-face treatment is on the rise for patients with MDD. To date, no study has examined whether sleep disturbance symptoms have an impact on bCBT treatment outcomes and whether it affects bCBT and treatment-as-usual (TAU) equally.

**Objective:**

The objectives of this study are to investigate whether baseline sleep disturbance symptoms have an impact on treatment outcomes independent of treatment modality and whether sleep disturbance symptoms impact bCBT and TAU in routine care equally.

**Methods:**

The study was based on data from the E-COMPARED (European Comparative Effectiveness Research on Blended Depression Treatment Versus Treatment-as-Usual) study, a 2-arm, multisite, parallel randomized controlled, noninferiority trial. A total of 943 outpatients with MDD were randomized to either bCBT (476/943, 50.5%) or TAU consisting of routine clinical MDD treatment (467/943, 49.5%). The primary outcome of this study was the change in depression symptom severity at the 12-month follow-up. The secondary outcomes were the change in depression symptom severity at the 3- and 6-month follow-up and MDD diagnoses at the 12-month follow-up, assessed using the Patient Health Questionnaire-9 and Mini-International Neuropsychiatric Interview, respectively. Mixed effects models were used to examine the association of sleep disturbance symptoms with treatment outcome and treatment modality over time.

**Results:**

Of the 943 patients recruited for the study, 558 (59.2%) completed the 12-month follow-up assessment. In the total sample, baseline sleep disturbance symptoms did not significantly affect change in depressive symptom severity at the 12-month follow-up (β=.16, 95% CI –0.04 to 0.36). However, baseline sleep disturbance symptoms were negatively associated with treatment outcome for bCBT (β=.49, 95% CI 0.22-0.76) but not for TAU (β=–.23, 95% CI −0.50 to 0.05) at the 12-month follow-up, even when adjusting for baseline depression symptom severity. The same result was seen for the effect of sleep disturbance symptoms on the presence of depression measured with Mini-International Neuropsychiatric Interview at the 12-month follow-up. However, for both treatment formats, baseline sleep disturbance symptoms were not associated with depression symptom severity at either the 3- (β=.06, 95% CI −0.11 to 0.23) or 6-month (β=.09, 95% CI −0.10 to 0.28) follow-up.

**Conclusions:**

Baseline sleep disturbance symptoms may have a negative impact on long-term treatment outcomes in bCBT for MDD. This effect was not observed for TAU. These findings suggest that special attention to sleep disturbance symptoms might be warranted when MDD is treated with bCBT. Future studies should investigate the effect of implementing modules specifically targeting sleep disturbance symptoms in bCBT for MDD to improve long-term prognosis.

## Introduction

### Major Depressive Disorder

Major depressive disorder (MDD) is among the most prevalent and debilitating psychiatric disorders [1-3], and it has been estimated to be the third leading cause of disability worldwide [3]. The MDD burden is particularly prominent in Western societies and poses an immense burden on society [1,3,4]. Although MDD, in many cases, can be successfully treated with evidence-based psychological and pharmacological therapies [5], some patients do not respond to treatment or do not receive adequate treatment [6]. To alleviate the burden of the disorder, we need to understand the mechanisms that may impact the lack of treatment response and develop strategies for broader dissemination of evidence-based psychological treatments [5,7].

### Depression and Sleep Disturbance

MDD is a heterogeneous condition with a variety of presentations and a broad constellation of associated symptoms. Sleep disturbances are core symptoms of MDD, covering mostly insomnia (difficulties in falling asleep, wakefulness after sleep onset, and/or waking up early and not being able to get back to sleep) and, to a lesser extent, hypersomnia (excessive daytime sleepiness and/or excessive total sleep time) [8]. Although insomnia and hypersomnia are common symptoms of MDD, they may also be diagnosed as independent psychiatric disorders [9]. Insomnia and mood disorders seem to have a bidirectional relationship, with insomnia increasing the risk for MDD, although MDD also increases the risk for insomnia symptoms [8]. This complex relationship seems to be founded on both common predisposing biological factors and cognitive and behavioral elements perpetuating both disorders, such as attentional biases and conditioning of arousal and negative affect in the bedroom [10]. Several studies have examined the impact of insomnia on the effect of MDD treatment and have consistently shown that insomnia affects the response to evidence-based treatment for depression, such as cognitive behavioral therapy (CBT) [11-14]. A total of 2 studies [12,14] reported that insomnia symptoms may double the risk for nonremission following depression treatment. In another study, patients exhibiting a limited response to treatment were more likely to present with recurring insomnia and depressive episodes, whereas optimal responders were consistently lower on insomnia measures [11]. In addition, a randomized controlled trial on older patients with MDD found that patients who presented with persistent insomnia symptoms, that is, reporting symptoms at baseline and the 3-month follow-up, were less likely to reach remission at the 6- and 12-month time points [13]. Furthermore, insomnia symptoms are among the most frequent residual symptoms following depression treatment [15-17]. Hypersomnia has not received the same attention as comorbid insomnia in MDD, perhaps because it is less common [18]. However, studies have found that persons with MDD who experience both hypersomnia and insomnia are more severely depressed compared with individuals with depression with only insomnia or no sleep disturbance symptoms [18,19].

### Digital Interventions

Digital interventions are often recommended as a tool to increase the dissemination of evidence-based psychological treatments [5,7]. Meta-analyses have shown that internet-based, guided self-help CBT (iCBT) is effective for treating depressive symptoms [20,21], and it may allow patients to circumvent some of the financial and structural barriers to psychological treatment [22]. However, iCBT is also affected by sleep disturbance symptoms. A cohort study on iCBT for depression in routine care showed that more sleep problems predicted higher depression by the end of treatment [23]. Furthermore, a randomized controlled trial on iCBT for chronic stress showed that the treatment effect was mediated by the reduction in insomnia symptoms [24]. Interestingly, some studies have shown that iCBT for insomnia also has significant effects on depressive symptoms [25,26], and in some cases, studies have also included insomnia management in the iCBT treatment manual for depression [27]. However, the added effect of doing so is yet to be documented.

As the self-help format in iCBT has a more rigid treatment structure, it might be more challenging to adapt the treatment to the individual’s symptom presentation. Standard CBT protocols often do not target sleep disturbance symptoms during treatment. However, within the face-to-face consultation, the therapist may have more flexibility to address other issues experienced by the patient. Perhaps even more so in routine care treatments, where therapists are not required to adhere strictly to a protocol as in a research project. The therapist could also favor another treatment approach that emphasizes sleep disturbance symptoms more than CBT. Digital interventions might lack this flexibility, when some or all of the treatment is delivered in a standardized format with predetermined exercises. One study also showed that although some patients feel empowered and safe in the iCBT format, others experience isolation and feel burdened by the limited therapist support [28].

### Blended CBT

Blended CBT (bCBT) might help bridge this gap between web-based and face-to-face treatment. In bCBT, patients receive a combination of web-based and face-to-face therapy. This may help therapists and patients adhere to treatment protocols while allowing for a different kind of flexibility during the physical consultations compared with the structured, guided self-help programs [29]. Face-to-face consultations in bCBT can alleviate the feelings of isolation that patients may experience in guided self-help, and it can help make the structured treatment content of CBT relatable and adaptable to the patient’s symptom profile [30]. However, no study has yet investigated the impact of sleep disturbance symptoms on digital interventions for MDD, including bCBT, compared with face-to-face treatments. As digital interventions are increasingly being offered to patients with MDD to augment the reach of evidence-based treatment, it is relevant to examine the association between the severity of sleep disturbance symptoms and treatment response to bCBT.

### Objectives

The objectives of this study are to investigate (1) whether baseline sleep disturbance symptoms have a negative impact on treatment outcomes for depression independent of treatment modality and (2) whether sleep disturbance symptoms impact bCBT and treatment-as-usual (TAU) in routine care equally.

On the basis of previous research, we hypothesized a priori that sleep disturbance symptoms would be negatively associated with treatment outcome independent of treatment modality.

## Methods

### Overview

This study was a substudy of the E-COMPARED (European Comparative Effectiveness Research on Blended Depression Treatment Versus Treatment-as-Usual; described in more detail in a study by Kleiboer et al [31]).

The trial was registered in clinical trial databases (ClinicalTrials.gov NCT02542891 [France], NCT02389660 [Poland], NCT02361684 [Spain], NCT02449447 [Sweden], and NCT02410616 [Switzerland]; other clinical databases: German Clinical Trials Register DRKS00006866 [Germany]; Netherlands Trials Register NTR4962 [the Netherlands], International Standard Randomised Controlled Trial Number registry ISRCTN12388725 [United Kingdom], and ClinicalTrials.gov NCT02796573 [Denmark]) and was conducted based on CONSORT (Consolidated Standards of Reporting Trials) [32].

### Study Design and Setting

The E-COMPARED trial was conducted as a 2-arm, parallel randomized controlled, noninferiority trial in nine European countries: France, Germany, the Netherlands, Poland, Spain, Sweden, Switzerland, the United Kingdom, and Denmark.

The trial was conducted in routine primary care facilities (sites: Germany, Poland, Spain, Sweden, and the United Kingdom) or specialized mental health care facilities (sites: France, the Netherlands, Switzerland, and Denmark).

### Eligibility Criteria

To be included in the study, patients had to (1) be aged ≥18 years, (2) fulfill Diagnostic and Statistical Manual of Mental Disorders-IV (DSM-IV) diagnostic criteria for MDD, and (3) exhibit minimal to severe symptoms of depression based on a score of ≥5 on a screening with the Patient Health Questionnaire-9 (PHQ-9; described in more detail in the *Assessment Instruments* section).

The exclusion criteria were as follows: (1) currently being at high risk for suicide (2) having fulfilled the DSM-IV diagnostic criteria for substance dependence, psychotic illness, bipolar affective disorder, or obsessive–compulsive disorder; (3) receiving psychological treatment at the time of enrollment for depression in primary or specialized mental health care facilities; (4) being unable to comprehend the spoken and written language of the country where the study was conducted (eg, English in the United Kingdom); (5) not having access to a computer with a fast internet connection; and (6) not having or not willing to carry a smartphone that is compatible with the mobile component of the intervention being offered.

### Randomization and Blinding

Eligible participants were randomized by a team of independent researchers (the randomization team) affiliated with the principal investigator organization (Vrije Universiteit Amsterdam) into two arms: TAU or bCBT. Randomization was performed at the individual level, stratified by country. The randomization team created the allocation scheme with a computerized random number generator (Random Allocation Software developed by Mahmood Saghaei) at an allocation ratio of 1:1. Block randomization with variable block sizes that varied between 8 and 14 allocations per block was applied.

None of the investigators or clinicians were aware of the randomization scheme, but blinding for treatment allocation was not possible owing to the nature of the treatment. Nonetheless, the outcome assessors were blinded.

### Treatment Arms

TAU was defined as the routine care that patients received when diagnosed with depression in the specific country and treatment setting where they were recruited. Thus, TAU varied between countries, between treatment settings, and among patients and included pharmacological treatment, psychotherapy, a combination of both, or none of the above (see Table 1).

The bCBT combined individual face-to-face CBT with CBT delivered through an internet-based treatment platform with mobile phone components (integrated either in the treatment platform or as a separate system). The core components of the bCBT treatment were the following: (1) psychoeducation, (2) cognitive restructuring, (3) behavioral activation, and (4) relapse prevention. These were delivered over 10 to 20 sessions. The ratio between the number of face-to-face sessions and the number of web-based modules varied according to practice in the participating countries. However, a minimum of 33% of the sessions were face-to-face, and a minimum of 33% of the sessions were provided through the web. In addition to the core CBT components, other components such as mindfulness, coping skills training, or problem solving could also be included (insomnia management was not included in this trial). However, they were not allowed to take up more than a quarter of the total treatment (no more than approximately 25% of the face-to-face and web-based sessions combined). This was done to prevent excessive heterogeneity in the treatment programs.

bCBT was provided by CBT therapists who received training on how to deliver the treatment. CBT therapists were (1) licensed CBT therapists, (2) CBT therapists in training working under the supervision of an experienced licensed CBT therapist in specialized mental health care facility, (3) licensed psychologists, or (4) psychologists in training working under the supervision of a licensed psychologist with a CBT orientation in primary care.

**Table 1:** Overview of blended cognitive behavioral therapy (bCBT) and treatment-as-usual (TAU) applied in each country.

Country	Platform for bCBT	Duration (weeks)	Web-based modules	FTF^a^ sessions	Sequencing	TAU
Germany	Moodbuster	10-13	10	5	Alternate	TAU from GP^b^
Sweden	Iterapi	10	6	4	Alternate	TAU from GP^c^
Netherlands	Moodbuster	20	10	9	Alternate	FTF TAU^d^
United Kingdom	Moodbuster	11	5	6	Alternate	FTF CBT
Spain	Smiling is fun^e^	10	8	3	1-4-1-4-1^f^	TAU from GP
France	Moodbuster	16	8	8	Alternate	FTF CBT
Switzerland	Deprexis^g^	18	9	9	Alternate	FTF CBT
Poland	Moodbuster	6-10	6	7	Alternate	FTF CBT
Denmark	NoDep^h^	12	6-8	6	Alternate	FTF CBT

^a^FTF: face-to-face.

^b^GP: general practitioner.

^c^Sweden also included psychotherapy clinics and student mental health care facilities. However, these are at the same level of care as GP.

^d^Psychotherapy (cognitive behavioral therapy, interpersonal psychotherapy, or supportive therapy), antidepressant medication, running therapy, or a combination of these.

^e^Additional module on coping skills.

^f^The sequence was as follows: 1 FTF session, 4 web-based modules, 1 FTF session, 4 web-based modules, and 1 FTF session.

^g^Additional modules on mindfulness, interpersonal skills, positive psychology, emotion-focused therapy, and childhood experiences.

^h^Two additional modules on restructuring of beliefs and management of rumination that clinicians could add to the web-based sessions if deemed necessary.

As can be seen in Table 1, none of the bCBT protocols included sleep disturbance symptoms interventions. However, we do not know to what extent sleep disturbance symptoms were included in the TAU protocol.

### Measures

Sociodemographic factors, MDD diagnoses, severity of depressive symptoms, and psychiatric comorbidities were measured at baseline. The clinical outcome measures used in this study were severity of depressive symptoms assessed at baseline and at the 3-, 6-, and 12-month follow-up and MDD diagnosis assessed at baseline and at the 12-month follow-up.

### Assessment Instruments

#### MDD Severity

The PHQ-9 was used to assess the severity of depression. It includes symptom domains of MDD based on DSM-IV (ie, sleep disturbance, sad mood, appetite and weight, concentration, self-criticism, suicidal ideation, interest, energy and fatigue, and psychomotor agitation and retardation). It consists of 9 items, each scored on a 0 to 3 scale, with the total score ranging from 0 to 27 and higher scores indicating more severe depression [33]. The PHQ-9 is frequently used in clinical trials to assess treatment outcomes and can be used in different patient populations such as those who receive primary care and specialized mental health care [34]. The PHQ-9 was used to screen for the presence of at least a minimum level of MDD severity (cutoff ≥5). Furthermore, the PHQ-9 total score was applied as the primary outcome. For this study, the 12-month follow-up was used as the primary end point. The 3- and 6-month follow-ups were the secondary end points.

#### MDD Diagnosis

The Mini-International Neuropsychiatric Interview (MINI) for DSM-IV is a structured interview probing the 17 most common psychiatric diagnoses using dichotomous questions requiring a yes or no response [35]. The MINI was used to establish MDD diagnoses as well as to screen patients for eligibility and detect comorbid psychiatric diagnoses. The MINI was also used to evaluate whether the patients still fulfilled the diagnostic criteria for an MDD diagnoses at the 12-month follow-up and was applied as a secondary outcome and secondary end point.

#### Assessment of Sleep Disturbance Symptoms

A total of four sleep disturbance items from the Quick Inventory of Depressive Symptomatology (QIDS)–Self-Report were used to measure sleep disturbance symptoms [36]: (1) difficulty falling asleep, (2) awake during the night, (3) waking up too early, and (4) sleeping too much. Each item was scored from 0 to 3, with higher scores indicating greater symptom severity. This approach to measure sleep disturbance symptoms has been validated previously for the 3 insomnia items [37]. However, the middle-answer categories (scores of 1 and 2) did not perform as well for items 2 and 3 [37]. Therefore, on the basis of previous research, a score ≥2 on an item was chosen to indicate the presence of that specific type of sleep disturbance [38]. A combination of any (or none) of the 4 types of sleep disturbance symptoms was possible.

### Analysis Plan

Characteristics of the sample at baseline were described using descriptive statistics and compared across groups using 2-tailed *t* tests for continuous variables and chi-square tests for categorical variables. If the continuous variables violated the assumption of normality, equivalent nonparametric tests were used (Wilcoxon signed rank test).

The primary analysis was performed using a linear mixed effects model with random intercept [39,40] with sites added as a random variable. The outcome variable was the difference in score on the PHQ-9 between baseline and at 12-month follow-up. The baseline sum score of the first 4 items on the QIDS–Self-Report pertaining to sleep disturbance symptoms was used as a predictor variable and was controlled for the baseline level of depression severity on the PHQ-9. To test for an effect of treatment modality (bCBT vs TAU), an interaction term between the sleep disturbance symptoms and treatment modality was added. A sensitivity analysis excluding item 3 in the PHQ-9 (pertaining to sleep disturbance) was also performed as this item could potentially confound the analyses.

Secondary analyses were also conducted with linear mixed effects models applying the same predictor and outcome variables as well as an interaction term, but at 3- and 6-month follow-up. In addition, an analysis using the same predictor variable and the presence versus absence of MDD diagnoses at 12-month follow-up as the outcome was conducted. This analysis was performed using a logistic mixed effects model.

All models were adjusted for the baseline level of depression on the PHQ-9 and sociodemographic variables (gender, marital status, highest education, and age).

All analyses followed the intention-to-treat principles according to CONSORT guidelines [41]. Missing values were handled by using all available data in mixed effects models. A double-sided significance level of .05 was applied. All calculations were performed using R version 3.6.3 [42]. Linear mixed effects models were fitted using the lme4 package [40].

### Ethical Considerations

Ethical approval for the trials was obtained locally in each country (Denmark: De Videnskabsetiske Komitéer for Region Syddanmark; S-20150150; France: Comité de protection des personnes, Ile de France V; 15033-n° 2015-A00565-44; Germany: Ethik Kommison DGPsychologie, Universitat Trier; MB 102014; The Netherlands: METC VUMC; 2015.078; Poland: Komisja ds. Etyki Badan Naukowych; 10/2014; Spain: Comision Deontologica/Comite Etico de Investigacion en Humanos; H1414775276823; Sweden: Regionala etikprovningsnamnden; 2014/428-31; Switzerland: Kantonale Ethikkomission Bern; 001/2015; United Kingdom: NRES Committee London-Camden and King’s Cross; 15/LO/0511). All participants provided written informed consent beforehand.

## Results

### Characteristics of the Sample

The sample consisted of 943 adult patients with MDD recruited from routine care across the 9 participating European countries. The mean age was 39 (SD 13) years, with mainly women included (644/943, 68.3%). The majority were either married or living together (517/943, 54.8%) and were well educated. At baseline, the sample, on average, presented with a moderate to severe degree of depression (mean PHQ-9 score 15.35, SD 4.77). There were no differences between the groups on any sociodemographic measures or on depressive or sleep symptomatology at baseline. The characteristics of the participants are summarized in Table 2.

**Table 2:** Comparison of characteristics across treatment modalities (N=943).

Characteristic			Total sample (N=943)	TAU^a^ (n=467)	bCBT^b^ (n=476)	*P* value^c^	
Age (years), mean (SD)			38.96 (13.09)	38.71 (13.08)	39.21 (13.1)	.56	
**Gender**							
	Female, mean (SD)		644 (68.3)	326 (69.8)	318 (66.8)	.36	
**Marital status** **,** **n (%)**							.27
	Single		314 (33.3)	155 (33.2)	159 (33.4)		
	Divorced		103 (10.9)	43 (9.2)	60 (12.6)		
	Widowed		9 (0.9)	6 (1.3)	3 (0.6)		
	Living together		206 (21.8)	111 (23.8)	95 (19.9)		
	Married		311 (32.9)	152 (32.5)	159 (33.4)		
**Highest level of education** **,** **n (%)**							.92
	Low		146 (15.5)	74 (15.8)	72 (15.1)		
	Middle		349 (37)	170 (36.4)	179 (37.6)		
	High		447 (47.4)	222 (47.5)	225 (47.3)		
**Trial site** **,** **n (%)**							.99
	Germany		173 (18.3)	87 (18.6)	86 (18.1)		
	Sweden		141 (14.9)	68 (14.6)	73 (15.3)		
	Netherlands		102 (10.8)	49 (10.5)	53 (11.1)		
	United Kingdom		101 (10.7)	52 (11.1)	49 (10.3)		
	Spain		127 (13.5)	63 (13.5)	64 (13.4)		
	France		105 (11.1)	54 (11.6)	51 (10.7)		
	Switzerland		50 (5.3)	24 (5.1)	26 (5.5)		
	Poland		84 (8.9)	42 (8.9)	42 (8.8)		
	Denmark		60 (6.4)	28 (5.9)	32 (6.7)		
**Psychometrics**							
	PHQ-9^d^, mean (SD)		15.36 (4.78)	15.38 (4.66)	15.34 (4.9)	.88	
	QIDS^e^ sum of sleep scores (items 1-4), mean (SD)		4.79 (2.42)	4.71 (2.31)	4.87 (2.52)	.31	
	**Insomnia items** **, n (%)**						
		Difficulty falling asleep	453 (48)	225 (48.2)	228 (47.9)	.99	
		Awake during the night	512 (54.3)	251 (53.7)	261 (54.8)	.76	
		Waking up too early	321 (34)	149 (31.9)	172 (36.1)	.18	
	**Hypersomnia item** **, n (%)**						
		Sleeping too much	135 (14.3)	65 (13.9)	70 (14.7)	.77	

^a^TAU: treatment-as-usual.

^b^bCBT: blended cognitive behavioral therapy.

^c^A 2-tailed *t* test was performed for continuous variables (age, PHQ-9, QIDS). Chi-square test was performed for categorical variables (gender, marital status, education, trial site and, insomnia and hypersomnia prevalence).

^d^PHQ-9: Patient Health Questionnaire-9.

^e^QIDS: Quick Inventory of Depressive Symptomatology.

### Observed Results

In this study’s sample of 943 patients, 764 (81%) reported at least one symptom of sleep disturbance symptoms at baseline. The most common symptom of sleep disturbance upon entering the study was midnocturnal insomnia (512/943, 54.3%), which was reported by more than half of the participants. Sleep onset insomnia (453/943, 48%) followed in close succession. Hypersomnia was markedly less prevalent than the rest (135/943, 14.3%).

**Figure 1:**
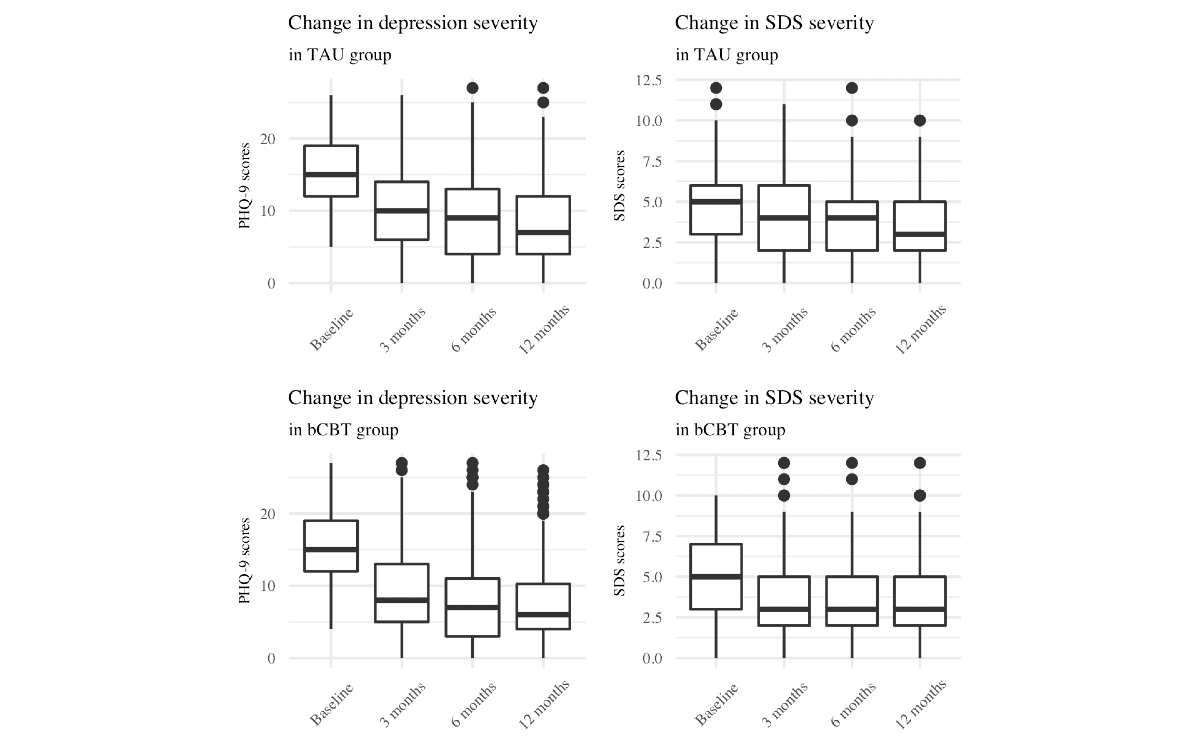
Changes in symptoms severity of depression and sleep disturbance symptoms from baseline to 12-month follow-up. bCBT: blended cognitive behavioral therapy; PHQ-9: Patient Health Questionnaire–9; TAU: treatment-as-usual.

As measured by the sum of the first 4 items in the QIDS pertaining to sleep, the level of sleep disturbance reduced over time, as seen in Table 3. In Figure 1, the levels of severity of depressive and sleep disorder symptoms are presented for the total sample and for each treatment condition.

**Table 3:** Observed means of sleep disturbance symptom severity.

Time point	Total sample, mean (SD)	TAU^a^, mean (SD)	bCBT^b^, mean (SD)
Baseline	4.79 (2.42)	4.87 (2.52)	4.77 (2.31)
3 months	3.89 (2.43)	3.83 (2.49)	3.94 (2.37)
6 months	3.61 (2.38)	3.52 (2.42)	3.69 (2.33)
12 months	3.28 (2.39)	3.27 (2.46)	3.30 (2.33)

^a^TAU: treatment-as-usual.

^b^bCBT: blended cognitive behavioral therapy.

### Primary Analysis

In the total sample, the level of sleep disturbance symptoms did not significantly predict the change in depression symptom severity from baseline to the 12-month follow-up (β=.16, 95% CI –0.04 to 0.36). However, there was a difference between the groups, with the effect of baseline sleep disturbance symptoms being nonsignificant in the TAU group (β=–.23, 95% CI –0.50 to 0.05), but being significant for the bCBT group (β=.49, 95% CI 0.22-0.76). When including an interaction term with group, the interaction was significant (β=.59, 95% CI 0.23-0.94), indicating a significant difference in how the change at 12 months was affected by baseline sleep disturbance symptoms between the groups. In a sensitivity analysis, we removed item 3 in the PHQ-9 (pertaining to sleep disturbances), which led to similar results. Therefore, we decided to continue using the full PHQ-9, given that it is the validated version and for the purpose of comparability. The results of the models are presented in Table 4.

**Table 4:** Associations between change in depression score and sleep score.

Outcome		All participants (N=943)				TAU^a^ (n=467)				bCBT^b^ (n=476)		
		Regression coefficient				Regression coefficient				Regression coefficient		
		n (%)	β^c^	Adjusted β^d^ (95% CI)	n (%)		β^c^	Adjusted β^d^ (95% CI)	n (%)		β^c^	Adjusted β^d^ (95% CI)
**Primary outcome**												
	Change in depression score at 12-months follow-up: sleep score at baseline, per 1 unit increase	558 (59.2)	.21	.16 (−.04 to .36)	274 (58.7)		.01	−.23 (−.50 to .05)	284 (59.7)		.35	.49 (.22 to .76)
	Difference between treatment conditions: SDS^e^ x condition	N/A^f^	.59	.59 (.23 to .94)	N/A		N/A	N/A	N/A		N/A	N/A
**Secondary outcomes**												
	Change in depression score at 3-months follow-up: sleep score at baseline, per 1 unit increase	748 (79.3)	.09	.06 (−.11 to .23)	379 (81.2)		.12	.07 (−.17 to .31)	369 (77.5)		−.07	.07 (−.16 to .32)
	Change in depression score at 6-months follow-up: sleep score at baseline, per 1 unit increase	657 (69.7)	.15	.09 (−.10 to .28)	319 (68.3)		.14	.03 (−.25 to .32)	338 (71)		.16	.12 (−.14 to .37)

^a^TAU: treatment-as-usual.

^b^bCBT: blended cognitive behavioral therapy.

^c^Adjusted for depression score at baseline.

^d^Adjusted for gender, age, marital status, highest education, and depression score at baseline.

^e^SDS: sleep disturbance symptoms.

^f^N/A: not applicable.

### Secondary Analyses

In line with the primary finding, baseline sleep disturbance symptoms severity did not significantly predict the presence of MDD diagnoses for the total sample as assessed by the MINI MDD module at 12-month follow-up (odds ratio [OR] 1.05, 95% CI 0.96-1.16). However, again, the effect was significant for the bCBT group (OR 1.18, 95% CI 1.02-1.38) and not for the TAU group (OR 0.94, 95% CI 0.82-1.08).

Owing to the diversity in interventions offered in the comparison group (TAU), we performed the same analyses for a subset of sites where the patients received face-to-face CBT as the comparator (United Kingdom, France, Switzerland, Poland, and Denmark). As was the case for all sites, the results of the subgroup were not significant for the total sample (β=.27, 95% CI −0.09 to 0.63), and only the bCBT group showed a significant effect of sleep disturbance symptoms (β=.69, 95% CI 0.19-1.19). However, the interaction term with group was nonsignificant (β=.56, 95% CI −0.07 to 1.22). This could be owing to the subgroup including fewer participants, thus widening the CI. Analyses of the presence of depression according to the MINI did not result in any significant results, which again may be caused by the smaller sample size.

The effect of sleep disturbance symptoms on the change in the severity of depressive symptoms was nonsignificant from baseline to the 3- and 6-month follow-up for both treatment formats, suggesting that sleep disturbance symptoms may not have an effect on short-term treatment gains.

Of the 4 items on sleep disturbance symptoms in the QIDS, 3 pertained to insomnia and 1 pertained to hypersomnia (see Table 2). Exploratory analyses were performed to examine whether there was a difference between the effect of hypersomnia and insomnia. When performing the same analysis as the primary analysis but excluding the hypersomnia item, it did not change the results. Furthermore, hypersomnia alone did not predict the treatment effect.

## Discussion

### Principal Findings

The objectives of this study were to investigate (1) whether baseline sleep disturbance symptoms have a negative impact on treatment outcomes independent of treatment modality and (2) whether sleep disturbance symptoms impact bCBT and TAU in routine care equally.

Overall, sleep disturbance symptoms did not have an impact on the treatment response. However, when comparing the treatment arms, a difference was observed in the effect of sleep disturbance symptoms on treatment outcome between bCBT and TAU. Higher baseline sleep disturbance symptoms indicated a decreased treatment response in the bCBT group, whereas TAU was unaffected. Interestingly, the effect of baseline sleep disturbance symptoms was only present at the 12-month follow-up. This finding points to a long-term effect of sleep disturbance symptoms on the course of depression following bCBT, even though treatment initially seems unaffected. Despite the effect being small, it was supported by the fact that sleep disturbance symptoms also significantly affected the risk of being diagnosed with MDD at the 12-month follow-up in the bCBT group.

However, in a secondary analysis of a subgroup of sites, we compared bCBT only with face-to-face CBT. This analysis showed that although sleep disturbance symptoms still significantly impacted bCBT, there was no significant difference between treatment arms in this subgroup. Not surprisingly, this could indicate that the face-to-face and blended CBT conditions were more similar in content and outcome compared with the remaining TAU formats. Perhaps the less structured TAU conditions were slightly more attentive to the presence of sleep disturbance symptoms, indicating a shortcoming in common CBT protocols for MDD.

This study found results that support previous research and results that contradict previous findings. In line with previous research and our hypothesis, bCBT was affected by sleep disturbance symptoms. However, contrary to this, TAU and the face-to-face CBT subgroup were unaffected by sleep disturbance symptoms.

The finding that sleep disturbance symptoms have a negative impact on bCBT represents an important addition to the existing literature on sleep disturbance symptoms and MDD treatment. It is in accordance with previous research showing an increased risk for MDD nonremission following face-to-face treatment [11,12,14], as well as studies showing that insomnia symptoms decrease treatment response in iCBT [23,24]. Hence, the results of this study extend the findings from previous research by confirming the association between sleep disturbance symptoms and treatment response for MDD in a new treatment format, bCBT.

Furthermore, the analyses showed that a higher endorsement of sleep disturbance items at baseline was associated with a lower treatment response at 12-month follow-up. This corroborates the findings of Pigeon et al [13] and Troxel et al [14]. However, although these authors only used a dichotomous measure of MDD (remission vs nonremission), the analyses of this study expanded this by showing an association between the severity of sleep disturbance symptoms and the degree of symptom alleviation at 12-month follow-up.

Although the long-term effect of baseline sleep disturbance symptoms on bCBT for MDD is in line with previous research [13], the finding of this study that treatment response at the 3- and 6-month follow-up was unaffected by sleep disturbance symptoms is not consistent with previous findings. Different explanations may apply. First, none of the previous studies investigated bCBT. This finding may be uniquely associated with bCBT. However, there are no clear indications as to why this should be the case. Second, neither MDD nor sleep disturbance symptoms has been measured uniformly in previous research. Notably, this study used sleep disturbance items from a depression scale (QIDS) to measure sleep disturbance symptoms. Although this method has been validated [37,43], it is not the most reliable measure of sleep disturbance symptoms. In the study by Troxel et al [14], subjectively measured sleep disturbance symptoms did not predict treatment outcome alone, but it did when coupled with an objective indicator. Another notable difference is the outcome measures used across these studies. As mentioned, previous research has generally focused on dichotomous outcomes [12-14], whereas this study used the PHQ-9 to measure the size of the treatment effect. If the MINI had been applied at the 3- and 6-month follow-up as well in this study, it would have been interesting to compare the dichotomous findings regarding nonremission at these time points. These differences might affect the comparability with the studies and therefore the findings of this study should not discount previous research.

The missing effect of sleep disturbance symptoms on TAU is also noteworthy. It might indicate that TAU practitioners were more attentive to sleep disturbance symptoms and implemented an intervention for sleep disturbance symptoms when necessary. However, this is still contrary to the study by Pigeon et al [13], who found a significant effect of insomnia on MDD treatment in routine care. Therefore, it may be worth noting that the study by Pigeon et al [13] is relatively old. It could be hypothesized that owing to the increased attention to sleep disturbance symptoms in the past years [8,10], routine care practitioners may treat patients reporting sleep disturbance symptoms differently today. This may help explain why TAU was unaffected by sleep disturbance symptoms in this study. However, it is highly speculative.

### Strengths and Limitations

Some strengths of this study are worth noting. Primarily, this study used a randomized and longitudinal design with multiple measurement points. Using this design, we can determine the extent to which bCBT compares with established effective treatments. Furthermore, we can determine the extent to which treatment effects are sustained following treatment.

Second, this study used a large, heterogeneous sample spanning 9 different European countries, lending the study great ecological validity. Furthermore, the comparison between TAU and bCBT provides clinically useful results, as TAU reflects the everyday treatments delivered by clinicians rather than the standardized treatments of a research trial. This part of the design could also be framed as a weakness because an efficacy study using only standardized protocols can be easier to interpret. However, this would have been at the cost of the finding’s generalizability [44], which was highly important in the E-COMPARED study [31].

Some limitations of the analyses of this study should also be considered. Primarily, the E-COMPARED study was not geared to investigate a research question regarding sleep disturbance symptoms, which is highlighted by the lack of a dedicated insomnia measure. However, as indicated by Manber et al [37], the items from the QIDS perform satisfactorily in discriminating the presence of sleep disturbance symptoms. Furthermore, we decided to pool hypersomnia and insomnia items to establish an aggregated score for sleep disturbance symptoms. Given the higher prevalence of insomnia, as was also evident in the sample of this study, this approach might be considered questionable. Focusing solely on insomnia symptoms would provide a clearer picture of what sleep disturbance symptoms entail. However, given that hypersomnia and insomnia symptoms may co-occur and have been associated with more severe depression in adolescents [18,19], capturing the full phenomenon seemed prudent.

This study provides important findings regarding the use of bCBT to treat MDD with marked sleep disturbance symptoms. When the treatment format moves from face-to-face to internet-based, the potential for broader dissemination generally comes at the cost of treatment flexibility. The analyses of this study indicated that sleep disturbance symptoms might affect the less flexible treatment format, bCBT, more than it affects TAU. However, given that this was a secondary analysis of a study that was not designed to test this hypothesis, the results should be interpreted cautiously. It will be important for future research to test whether these findings are supported when using dedicated sleep disturbance measures. Nonetheless, it would also be of interest to see if treatment outcomes in bCBT can be improved by using web-based add-on sessions for sleep disturbance symptoms. Previous research has shown that iCBT can effectively treat sleep disturbance symptoms [25,45-48], and there is a call for research on sequential or concomitant treatment of MDD and sleep disturbance symptoms [49]. Finally, it is of interest to investigate whether these findings extend to other forms of diagnostic complexity. Future studies should compare bCBT with face-to-face therapy for patients with MDD with other common comorbidities such as alcohol use or anxiety disorders.

### Conclusions

Baseline sleep disturbance symptoms may have a negative impact on long-term treatment outcomes in bCBT for MDD. TAU seems to be unaffected by the severity of the baseline sleep disturbance symptoms. This suggests that extra attention to comorbidity such as sleep disturbance symptoms, which was investigated here, might be important when treating depression with bCBT in routine care.

Future research should investigate whether there is an improved treatment outcome in bCBT for MDD with comorbid sleep disturbance symptoms when using add-on modules targeting sleep disturbance symptoms.

## Abbreviations

bCBT: blended cognitive behavioral therapy
CBT: cognitive behavioral therapy
CONSORT: Consolidated Standards of Reporting Trials
DSM-IV: Diagnostic and Statistical Manual of Mental Disorders-IV
E-COMPARED: European Comparative Effectiveness Research on Blended Depression Treatment Versus Treatment-as-Usual
iCBT: internet-based cognitive behavioral therapy
MDD: major depressive disorder
MINI: Mini-International Neuropsychiatric Interview
OR: odds ratio
PHQ-9: Patient Health Questionnaire-9
QIDS: Quick Inventory of Depressive Symptomatology
TAU: treatment-as-usual
